# A meta-analysis investigating the relationship between inflammation in autoimmune disease, elevated CRP, and the risk of dementia

**DOI:** 10.3389/fimmu.2023.1087571

**Published:** 2023-01-27

**Authors:** Joseph Cooper, Ylenia Pastorello, Mark Slevin

**Affiliations:** ^1^ Department of Life Sciences, Manchester Metropolitan University, Manchester, United Kingdom; ^2^ George Emil Palade University of Medicine, Pharmacy, Science and Technology, Targu Mures, Romania

**Keywords:** C-reactive protein, inflammatory disease, dementia, autoimmune, meta - analysis

## Abstract

Alzheimer’s Disease (AD) represents the most common type of dementia and is becoming a steadily increasing challenge for health systems globally. Inflammation is developing as the main focus of research into Alzheimer’s disease and has been demonstrated to be a major driver of the pathologies associated with AD. This evidence introduces an interesting research question, whether chronic inflammation due to pathologies such as inflammatory bowel disease (IBD) and rheumatoid arthritis (RA) could lead to a higher risk of developing dementia. In both IBD and RA, increased levels of the inflammatory biomarker C-reactive protein (CRP) can be highlighted, the latter being directly implicated in neuroinflammation and AD. In this meta-analysis both the association between chronic inflammatory diseases and elevated levels of CRP during midlife were investigated to examine if they correlated with an augmented risk of dementia. Moreover, the association between increased CRP and modifications in the permeability of the Blood Brain Barrier (BBB) in the presence of CRP is explored. The results displayed that the odds ratio for IBD and dementia was 1.91 [1.15-3.15], for RA it was 1.90 [1.09-3.32] following sensitivity analysis and for CRP it was 1.62 [1.22-2.15]. These results demonstrate a higher risk of dementia in patients presenting chronic inflammation and that exists an independent association with high CRP in midlife. This paper builds on published research that suggest a critical role for CRP both in stroke and AD and provides an analysis on currently published research on multiple diseases (IBD and RA) in which CRP is raised as well as chronically elevated. CRP and the associated risk of dementia and further research indicated that the monomeric form of CRP can infiltrate the BBB/be released from damaged micro-vessels to access the brain. This meta-analysis provides first-time evidence that chronic elevation of CRP in autoimmune diseases is directly associated with an increased risk of later development of Alzheimer’s disease. Therefore, greater priority should be provided to the effective control of inflammation in patients with chronic inflammatory or autoimmune conditions and further long-term assessment of circulating CRP might inform of an individual’s relative risk of developing dementia.

## Introduction

Worldwide, dementia represents one of the most prominent health crises accounting for 50 million patients suffering from the disease, with an additional 10 million being diagnosed each year with around 115 million estimated to be affected by 2050. Alzheimer’s Disease (AD) constitutes 60-70% of the total cases (1). AD is characterised by the formation of amyloid beta plaque and intercellular neurofibrillary tangles (2) and diagnosis relies on neuroimaging and biomarkers.

Chronic inflammatory diseases represent a steadily increasing challenge for health systems globally, with inflammation playing a key-role in the vast majority of chronic pathologies. The two chronic inflammatory diseases examined in this work are Inflammatory Bowel Disease (IBD), which includes Crohn’s disease (CD) and Ulcerative Colitis (UC), and Rheumatoid Arthritis (RA). IBD is defined as a chronic inflammation of the gastrointestinal tract (GI tract): Other chronic immune disease including Giant Cell arteritis (GCA) and Ankylosing Spondylitis and Lupus, were not included since there is insufficient literature or clinical studies available to conduct meta or systemic analysis. CD can affect any of its segments whereas UC only affects the large intestine (3). RA is an autoimmune disease responsible for articular pain, swelling and stiffness; risk factors include family history and female gender (4). Both IBD and RA can be characterised by chronically raised levels of C-reactive protein (CRP) which is a widely used biomarker of inflammation. CRP is a pentameric protein synthesised in the liver in response to interleukin-6 and plays a pivotal function in the inflammatory response (5). In addition, it is currently acknowledged that irreversible dissociation of this molecule into monomers is accompanied by its biological activation, and stimulation of inflammatory processes including potentially neurodegeneration (6).

In our attempts to comprehend this complex disease we noted that inflammation has been extensively reported as a key-factor in the development of dementia. Therefore, we aimed to investigate whether there might be an association between chronic inflammation, defined by measurement of circulating CRP in chronic inflammatory diseases, and a higher risk of developing dementia or cognitive decline.

## Methods

### Search strategy-linking chronic inflammatory diseases with dementia

Our research was performed in PubMed and Web of Science including the search terms ‘Inflammatory Bowel disease’, ‘IBD’, ‘Rheumatoid Arthritis’, ‘RA’, ‘mild cognitive impairment’, ‘MCI’, ‘cognitive impairment’, ‘Alzheimer’s Disease’, ‘AD’, ‘dementia’, ‘C-reactive protein’, ‘CRP’ and ‘inflammatory biomarkers’.

Studies investigating the association between inflammatory bowel disease, rheumatoid arthritis and dementia, Alzheimer’s disease or cognitive impairment were included. Longitudinal population-based studies and studies comparing chronic inflammatory disease patients with either IBD or RA with healthy controls (Case control studies) were comprised. All articles have been formulated in English language and have required accessibility to mine the data.

### CRP and dementia

Studies that examined the association between raised levels of serum CRP during an individual’s life and the incidence of dementia, AD or cognitive impairment (identified through clinical diagnosis or cognitive testing) were encompassed. Longitudinal population- based studies and studies comparing participants with elevated CRP levels were evaluated. Other inclusion criteria were represented by papers elaboration exclusively in English language and required accessibility for data mining.

### Study selection

**Figure 1:**
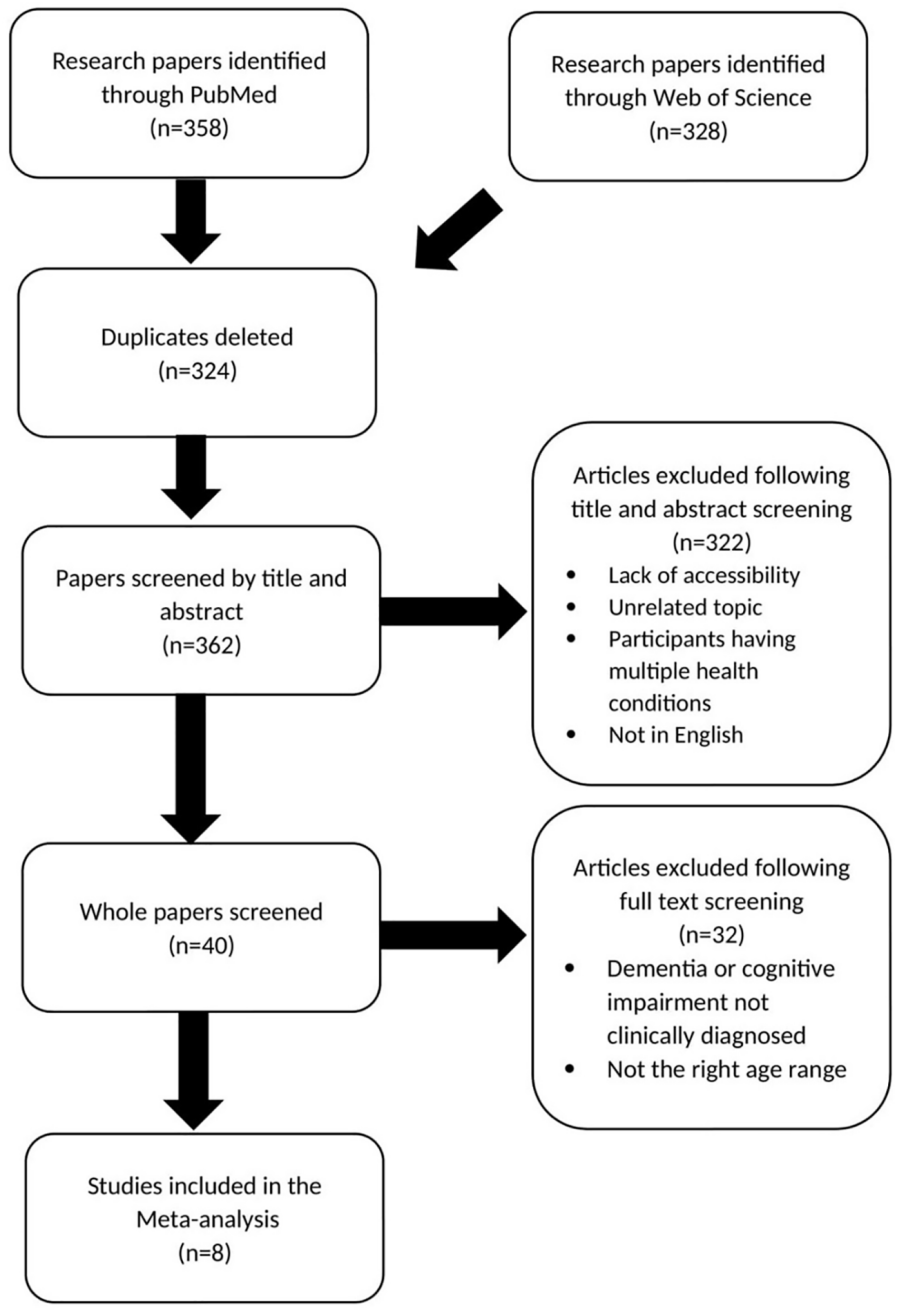
Prisma chart showing paper selection for meta-analysis investigating IBD and RA association with risk of dementia.

**Figure 1** shows the method by which studies were selected for meta-analysis for IBD/RA association with risk of dementia (7–14).

**Figure 2:**
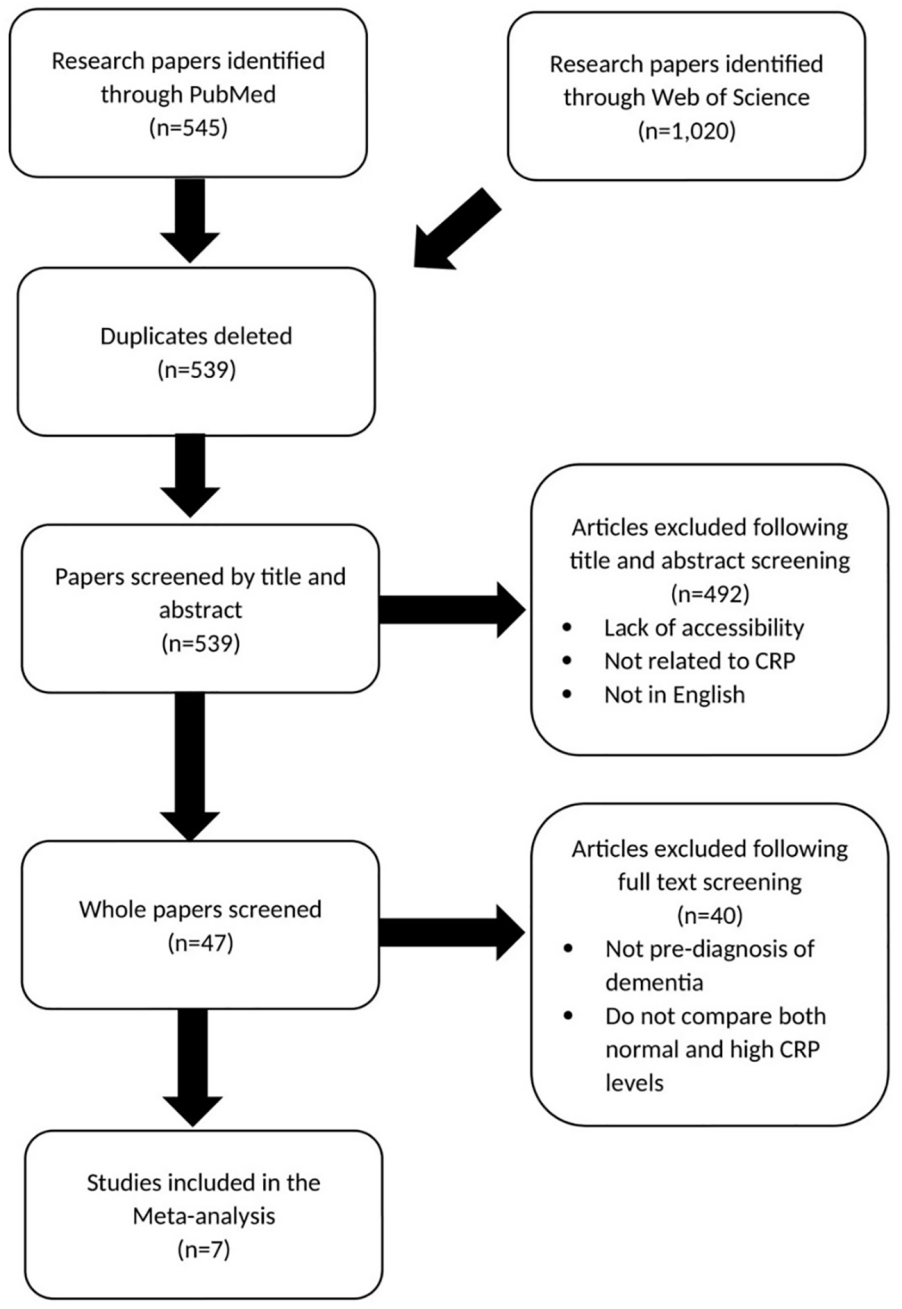
Prisma chart showing paper selection for meta-analysis investigating CRP association with dementia risk.

**Figure 2** shows the method of study selection for the meta-analysis investigating the relationship between midlife elevated CRP and the risk of dementia and cognitive impairment (15–21).

### Data collection

From each paper the author, title, and date of publication was recorded and subsequently the odds ratio, the standard deviation and number of participants were extracted.

### Meta-analysis

Two meta-analyses were performed, one exploring the association between chronic inflammatory diseases (IBD and RA) and dementia and another examining the link between serum CRP and dementia. The research papers included in **Figures 1**, **2**, formed the basis of the meta-analysis. The meta-analysis was conducted imputing inverse variance data and was performed with random effects and a 95% CL. Both forest plots and funnel plots were produced. All analysis was conducted using Review Manager 5.4.1.

## Results

### Inflammatory bowel disease and dementia

**Figure 3:**
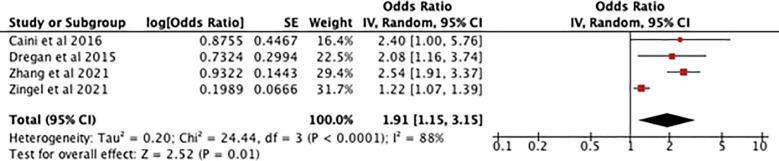
forest plot showing the results of the meta-analysis exploring the relationship between IBD and risk of dementia. Positive values indicate that dementia and cognitive decline are more prevalent in IBD patients, with the P value showing its statistical significance (7–10).

**Figure 3** shows an odds ratio of 1.91 [1.15-3.15] for the association between IBD and dementia and cognitive impairment meaning dementia is 1.91 times more prevalent in patients with IBD. The heterogeneity was I^2^ = 88%. The test for overall effect presented [Z=2.52, P=0.01] meaning the result was statistically significant and the null hypothesis can be rejected. Subsequent sensitivity analysis found that if the study by Zingel et al. (10) was given no weight in the meta-analysis the I^2^ went down to 0%. Because of high heterogeneity a sensitivity analysis was conducted which found that when Zingel et al. (10) was removed, I^2^ was reduced to 0% with an odds ratio of 2.44 [1.91-3.12] and a test for overall effect [Z=7.16, P=0.00001].

### Rheumatoid arthritis and dementia

**Figure 4:**
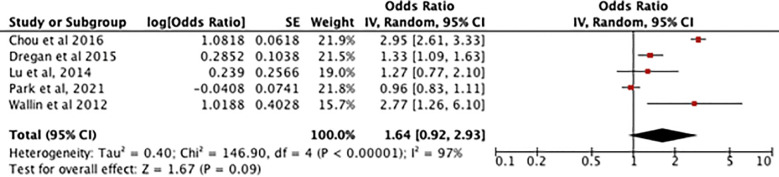
Forest plot showing the results of meta-analysis investigating the relationship between RA and risk of dementia. Positive odds ratio values indicate that dementia is more prevalent in rheumatoid arthritis patients, with the P value showing its statistical significance. (8, 11–14).

**Figure 4** shows an odds ratio of 1.64 [0.92-2.93] for the association between RA and dementia/cognitive impairment, meaning dementia is 1.64 times more prevalent in patients with RA. The heterogeneity was I^2^ = 97%. The test for overall effect was [Z=1.67, P=0.09] meaning the result is not statistically significant so the null hypothesis cannot be rejected.

**Figure 5:**
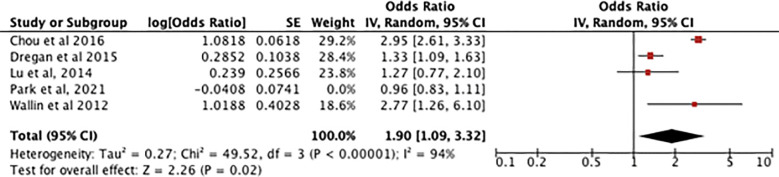
shows a sensitivity analysis of the association between RA and dementia but with an outlier (Park et al,2021) giving 0% weight.

**Figure 5** showing the sensitivity analysis of the RA meta-analysis excluding the study by Park et al. (13) displays an odds ratio of 1.90 [1.09-3.32] for the association between RA and dementia, meaning dementia is 1.90 times more likely to occur in patients with RA. The heterogeneity was I^2^ = 94%. The test for overall effect showed [Z=2.26, P=0.02] meaning the result is statistically significant and the null hypothesis can be rejected.

### C-reactive protein and dementia

**Figure 6:**
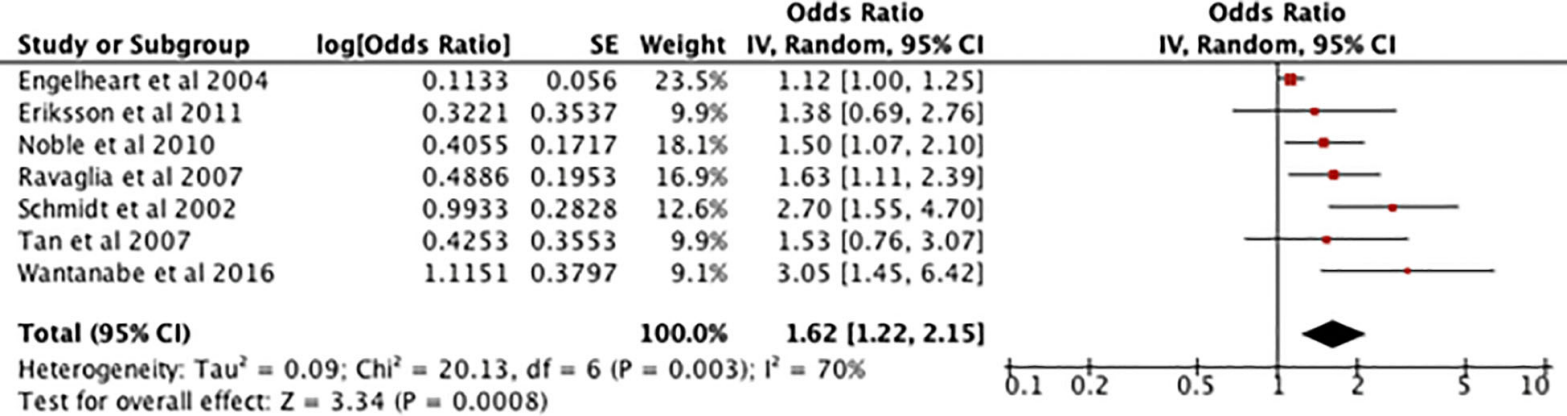
Forest plot showing the results of a meta-analysis investigating the relationship between midlife raised CRP serum levels and dementia. A positive odds ratio value indicates that dementia is more prevalent in participants who have had raised serum CRP and the P value shows its statistical significance (15–21).

**Figure 6** shows an odds ratio of 1.62 [1.22-2.15] for the association between high serum CRP in midlife and development of dementia, meaning dementia is 1.62 times more prevalent in patients with midlife elevated serum CRP. There is a I^2^ of 70% and [Z=3.34, P=0.0008] indicating that the results are highly significant so the null hypothesis can be rejected.

Because of a high heterogenicity, a subsequent sensitivity analysis was performed which found that, when Engelhart et al. (15) was removed, the results were 1.76 [1.40 - 2.22], I^2^ = 17% with a test of overall effect displaying [Z=4.86, P=0.00001].

## Discussion

The meta-analysis performed on IBD and dementia highlighted an increased risk of developing dementia in patients with IBD and this was found to be statistically significant. However, there was a high I^2^ which suggested there was significant heterogeneity: subsequent sensitivity analysis detected that if the study by Zingel et al. (10) was given no weight in the meta-analysis the I^2^ went down to 0%. Initially, the association between RA and dementia was not regarded to be statistically significant however, a subsequent sensitivity analysis which removed one of the included studies, found the results to be statistically significant. A cross-sectional study carried out by McDowell et al. (22) confirmed the high prevalence of cognitive impairment in RA being partly driven and further accelerated by chronic systemic inflammation. Cognitive impairment per se appears to be associated with higher RA disease activity, with increased CRP levels representing one of the core components of the 28-joint Disease Activity Score (DAS-28) and a widely used marker of RA activity and progression.

Nevertheless, augmented CRP levels cannot be universally regarded as a direct reflection of RA disease activity, given the considerable patient population who are subject to flares but displays CRP levels in the normal ranges (23). Mason et al. (24) raised the intriguing theory that by controlling the systemic inflammation seen in RA, deceleration in deterioration of cognitive function could be achieved. Treatment of RA consists of short-term glucocorticoids, long-term Disease Modifying Anti-Rheumatic Drugs (DMARD) therapy and, in case of persistent elevated disease activity, biological agents such as Tumor Necrosis Factor Inhibitors (TNFi). Interestingly, several cases of cases of “Steroid Dementia”- development of dementia following intake of corticosteroid- one of which in a RA patient, were reported with the psychiatric symptoms being attributed to the high-dose treatment.

In respect of IBD, Liu et al. (25), produced a recent updated meta-analysis showing a significant link, identified in previous cohort studies, with development of dementia, that was independent of age and sex, although not specifically AD. Our current study based upon this foundation, looked to identify a possible mechanism through examination of concomitant raised levels of CRP, since recently this has been shown to be closely associated with neurodegenerative progression (see later explanation).

It is broadly acknowledged that patients undergoing corticosteroids treatment present an increased risk to develop mental disturbances (26). DMARDs appears to provide benefits to cognitive functions by controlling the systemic inflammatory response but further studies are required (22). Exposure to TNFi agents, reported to be successful also in treatment of IBD, is associated with decreased risk of AD among RA patients. Furthermore, the incidence of dementia among TNFi patients has been proven to be lower compared with the rate observed in the conventional synthetic DMARDs category (24).

The meta-analysis investigating levels of CRP and rates of dementia displayed that the risk of developing dementia was statistically significantly higher with elevated serum levels of CRP. A recent systematic review and meta-analysis by Long et al. (27) assessed that increased CRP values in peripheral blood, as an indicator of chronic systemic inflammation, cannot anticipate later cognitive impairment but may highlight an augmented risk of conversion to dementia. This corroboration could provide a link between other meta-analyses examining IBD and RA as both are characterised by raised CRP levels.CRP is also prevalent in many other inflammatory conditions, such as GCA, a form of vasculitis affecting the blood vessels of the head, neck, and upper limbs; this causes arterial stenoses and potentially occlusions. The main symptoms of GCA include headaches, jaw claudication, reduced visual acuity, diplopia, and even acute visual loss. Because of the inflammatory nature of this pathology, inflammatory biomarkers such as CRP are present in higher levels in most patients (28). Therefore, it could be speculated (although published data is not yet available to prove this hypothesis) that other diseases causing elevated CRP may increase the risk of dementia.

Other studies have also indicated that chronic inflammation can cause brain damage, including one study that followed 1,500 participants for over 21 years monitoring the CRP levels regularly. At the final appointment each patient underwent a magnetic resonance imaging (MRI) which highlighted how the 90 participants, identified as having chronic inflammation, also had the most extended damage in cerebral white matter. This points towards a role of chronic inflammation in cerebral health (29). Another study found that elevated CRP levels correlated with a decreased brain function by measuring the regional cerebral blood flow (30). In a longitudinal study which comprised participants aged over 90 years, it was demonstrated that elevated CRP values were associated with increased odds of all-causes dementia (31).

In the context of chronic inflammatory diseases, the arising dilemma concerns the dynamics related to the penetration of the BBB by systemic CRP, which proceeds to infiltrate the brain and potentially cause dementia. One plausible explanation was provided by Slevin et al. (6), where the group showed how mCRP increased vascular monolayer permeability and gap junctions. This could represent an elucidation on the mechanisms of chronic inflammation leading to dementia, through the associated mCRP increasing vascular permeability which could allow concurrent infiltration of CRP and inflammatory cytokines into the cerebral matter, perpetuating neuroinflammation and neurodegeneration. Hsuchou et al. (32) conducted a study using radioactively tagged CRP to test BBB permeability in mice and found that, whilst low levels of CRP did not increase paracellular permeability, high doses did. This latter research in the context of our study appears to be of great relevance as chronic inflammatory diseases are often characterised by high levels of CRP, providing further evidence for the possible increase in BBB permeability in chronic inflammation. Nonetheless, these results originate from animal studies, thus some caution should be taken when relating them to humans.

One possible criticism to the argument stating that mCRP increases vascular permeability consists in the evidence that mCRP is relatively insoluble and precipitates at the site of inflammation, therefore is not easily transported in the systemic circulation. However, studies exploring the role of mCRP in other diseases including myocardial infarction and peripheral arterial disease highlighted that mCRP can circulate by binding to cellular microparticles and leukocytes, which aid the conversion of CRP into mCRP (33, 34). It is known that in inflammatory states cellular microparticles are produced through various mechanisms, including oxidative stress. It has been shown that in Crohn’s disease, these microparticles are increased (35) as well as in RA (36). Hence, this could represent the association between elevated CRP levels during chronic inflammatory conditions, increased BBB permeability, and subsequent infiltration of CRP in the brain as potential cause of Alzheimer’s disease and dementia.

At the site of inflammation, CRP can dissociate into its monomeric form which is considerably less soluble. mCRP can accumulate at sites of vascular injury and inflammation and, in the case of ischemic stroke, has been proven to be present in the infarcted tissue in notable quantities (37). Therefore, mCRP could represent one of the responsible factors for the high dementia rates in stroke patients. A study investigating the effects of mCRP injected into the brains of mice showed that, within one month, the mice displayed behavioural and cognitive decline. In post-mortem studies of stroke patients with AD, there was co-localization of mCRP with amyloid plaques and tau like fibrils. These findings suggest that mCRP may directly contribute to AD (6). This research could point to a therapeutic target for patients recovering from ischemic stroke, following thrombosis, by modulation of mCRP and concurrently limiting its potential to promote vascular neurodegeneration and Alzheimer’s disease.

Further evidence that mCRP is involved in neuroinflammation and consequently AD was disclosed by a research examining the immunohistochemical staining of brain tissue samples of AD patients. The study exhibited that mCRP staining surrounded the abnormal brain tissue with a vascular disturbance and histological evidence of a strong local immune response. Similarly, mCRP staining was identifiable around leaky blood vessels and positively stained cells were localised in the abnormal tissue. Evidence of the key-role of mCRP in neuroinflammation in AD patients was also demonstrated by its co-localization with CD68 and IL-1β (38). This suggests that neuroinflammation and brain injury is strongly associated with co-localization of mCRP, implying its remarkable contribution in the pathophysiology of AD.

## Conclusions

This meta-analysis provides first-time evidence that chronic elevation of CRP in autoimmune diseases may be associated to an increased risk of later development of neuroinflammation and possibly dementia. Since there is now evidence suggesting that chronically raised CRP levels are associated with increased risk of dementia and also other serious disease including development of premature coronary artery disease (39), it would be important to ascertain if the length of chronic exposure was related to the ultimate extent of damage, morbidity and mortality.

So far nothing has been reported in the literature, however, recent published work has highlighted the critical importance of circulating levels of the monomeric form of CRP in relation to risk of development of carotid atherosclerotic plaques, and that this was not significantly correlated with total systemic circulating native CRP but only LDLc (40). Both the consequences of long-term exposure to raised CRP together with the mechanisms responsible for production of the particulate circulating mCRP should be investigated with some urgency as this appears to be a key component and promoter of disease progression.

Long-term assessment of circulating CRP/mCRP can be considered as a precious tool to enable estimation of an individual’s relative risk of developing dementia. Therefore, therapeutic strategies aimed to control and minimize the inflammatory states of patients with chronic inflammatory or autoimmune conditions is of utmost importance in order to limit the progression of neurodegeneration.

## Author contributions

All authors contributed to the article and approved the submitted version.
